# Stress-Reducing Effect of a 50 Hz Electric Field in Mice after Repeated Immobilizations, Electric Field Shields, and Polarization of the Electrodes

**DOI:** 10.3390/biology11020323

**Published:** 2022-02-17

**Authors:** Shinji Harakawa, Takaki Nedachi, Toshikazu Shinba, Hiroshi Suzuki

**Affiliations:** 1 Bio-Self-Regulating Science Laboratory, Obihiro University of Agriculture and Veterinary Medicine, Obihiro 0808555, Japan; hisuzuki@obihiro.ac.jp; 2 Hakuju Institute for Health Science, Tokyo 1510063, Japan; nedachi@hakuju.co.jp; 3 Department of Psychiatry, Shizuoka Saiseikai General Hospital, Shizuoka 4228527, Japan; t156591@siz.saiseikai.or.jp

**Keywords:** stress, endocrine response, extremely low frequency, electrical stimulation, physical therapy

## Abstract

**Simple Summary:**

With the increasing demand for electricity and electrical equipment, humans are routinely and unintentionally exposed to electric fields (EFs). Although no considerable adverse effects of EF exposure have been observed, slight physiological effects are known to occur. Additionally, there are methods and devices that expose subjects to EF for medical purposes. The mechanism of the biological effects of EF has not been identified, because the effects are not strong and may involve the physical properties of EFs, which are invisible and easily disturbed by obstacles. In a simple and short experiment using mice, we found that EF has an inhibitory effect on glucocorticoid (GC) responses. The experiment’s reproducibility was almost 100%. We tried to improve the understanding of the biological effects of EF by structuring our observations of the stress-reducing effects under different conditions in the system. We found that the inhibitory effect on the GC response was attenuated by EF shielding. We compared the effects of EF shielding between the head and abdomen, and found that the effects of EF were attenuated in both conditions, but might be more attenuated when the head was shielded. Thus, it appears that the area where the EF is distributed and the body part are important for the biological effects of EF. Two experiments with different conditions were performed. These results will help advance the current understanding of the effects of EF on stress systems.

**Abstract:**

In BALB/c mice, immobilization-increased plasma glucocorticoid (GC) levels are suppressed by extremely low frequency (ELF) electric fields (EF). The aim of this study was to advance our understanding of the biological effects of ELF-EF, using its suppressive effect on the GC response. Mice were exposed to a 50 Hz EF of 10 kV/m via a parallel plate electrode and immobilized as needed. We examined the suppressive effect of ELF-EF on GC level change after repeated immobilizations, electrode polarization, and EF shielding of different portions of the mouse body parts. Additionally, bodyweight changes owing to stress and EF were examined. Immobilization-induced reduction in the plasma GC levels was reproduced in mice with stress and EF exposure, regardless of the stress episode numbers and electrode polarization. Furthermore, when the head of mice was shielded from the EF, the suppressive effect was possibly relatively lower than that when the abdomen was shielded. The bodyweight of the mice decreased for 3 days after immobilization before recovering; ELF-EF did not affect the bodyweight. Thus, to elicit the biological effects of the EF, not only the size of the area where the EF is distributed but also the area where the field is distributed should be important. The results also confirmed the stableness of the present experimental system, at least in terms of the stress-reducing effect. In addition, the restriction in this study caused weight loss, but ELF-EF was not considered to affect it. The results improve the understanding of the biological effect and medical applications of ELF-EF.

## 1. Introduction

The widespread use of electricity in domestic and industrial settings highlights the importance of investigating the biological effects of extremely low-frequency (ELF) electric field (EF), particularly at 50 and 60 Hz power-line frequencies [1,2,3,4]. ELF-EF has been applied in clinical treatment and health maintenance in medical facilities and homes [2,5,6,7,8,9]. Several established and familiar treatment methods and devices involving EF have been used globally in various treatments, including transcutaneous electric nerve stimulation and vagal nerve stimulation, as therapies for depression, epilepsy, dementia, and pain. Although the electric current induced in the body and the perception of EFs by the skin surface could trigger cellular and humoral responses in certain organisms, our understanding of the mechanism of the biological effects induced by EF is not sufficient [3,10,11,12,13,14,15,16,17,18,19]. This is because the effect of EF is not clear; furthermore, the physical properties of EF, which is invisible and easily disturbed by obstacles, may be involved. Therefore, understanding the biological effects of ELF-EF is useful from both perspectives, and it is necessary from a human health perspective. In addition, for ELF magnetic fields, the main interaction mechanism is the induction of EF in the body that may have biological or health effects depending on its strength. However, for power-frequency ELF-EFs, the coupling to the induced EF in the body is much weaker, and surface electric charge effects on the body might be a more prominent mechanism [20].

In order to assess such unclear biological effects, a reproducible evaluation system is needed. Previously, we reported some biological effects of ELF-EF exposure for alleviating the pain of musculoskeletal or of undefined origin and insomnia [7,8,21]. The bone density of humans exposed to EF therapy is higher than that of age-matched humans, and the bone density increased as the duration of EF treatment increased [21]. Moreover, ELF-EF can modulate energy metabolism [22], cell signaling pathways [23,24,25,26], the endocrine system [22,27], the immune system [28], and behavioral control [29]. However, to study the biological effects of EF, which is easily affected by external disturbances, an evaluation system that is simpler and requires a shorter time is preferable.

An evaluation system that uses the glucocorticoid (GC) response of mice to EF generated using parallel plate electrodes has been developed. Using the system, we observed the suppressive effect of EF exposure on the elevation of stress hormones during restraint, and the reproducibility of the effect of EF was almost 100% [22]. Corticosterone, the main GC produced by the adrenal gland, regulates the expression of the corticotropin-releasing factor gene or the proopiomelanocortin gene [30]. An increase in the glucocorticoid level can accompany an acute stress response as an adaptive mechanism [31,32]. In our system, the procedure of immobilization and EF treatment was completed within 30–60 min, and it seems to be a candidate of the reproducible evaluation system to assess unclear biological effects of EF. The effect of EF was found to be dependent on the intensity (kV/m) and exposure duration [33], as well as the configuration of the EF exposure system [34]. For example, the immobilization-induced GC levels were significantly decreased in mice exposed to an EF of 1 kV/100 mm for 60 min, but not in mice exposed to 0.5 kV/50 mm or 2 kV/200 mm, although these systems generate a similar EF strength [34]. Additionally, the suppressive effect of EF exposure is dependent on the body surface area exposed to the EF [35], and it is independent of sex and age [36]. The evaluation system used in this study can elucidate the exact biological effects of power-line-frequency EF both qualitatively and quantitatively. As the relationship between GC reduction and ELF-EF is better understood, not only as a biohazard but also as a developable component of numerous medical applications of ELF-EF, it can be studied based on GC’s wide range of functions [37,38,39,40,41,42].

The bone density of humans using EF for therapeutic purposes is higher than that of humans of the same age, and the bone mineral density increases with an increase in the duration of EF treatment [21]. As osteoporosis is caused by the excessive production of glucocorticoids [43], the regulation of GC levels by ELF-EF may be involved in the mechanism. Furthermore, ELF-EF has been reported to be effective in alleviating musculoskeletal and unexplained pain and insomnia [8]; GCs modulate inflammation [38,39,40,41,42], suggesting an effect of ELF-EF on stress and tissue damage involving inflammatory events. A better understanding of the relationship between plasma GC concentrations and ELF-EF may lead to the discovery of additional medical applications of EF.

In this study, we tried to improve the understanding of the biological effects of EF by structuring our observations of the stress-reducing effects under different conditions in the system. The dependency of the suppressive effect of ELF-EF after repeated immobilization of the mice, switching the polarization of the electrodes, and EF shielding applied to different areas of the mice was tested.

## 2. Materials and Methods

### 2.1. Animals

Eight-week-old male BALB/c mice (*Mus musculus*) were purchased from Charles River Japan (Kanagawa, Japan) and maintained in a pathogen-free environment at 24 ± 1 °C and 50% ± 10% humidity with daily artificial illumination (12:12 h light/dark cycle with lights on from 07:00 to 19:00). The animals had free access to standard laboratory chow (CE-2; CLEA, Tokyo, Japan) and water, except for the period during EF exposure and immobilization. The adaptation period after the arrival of animals was 2 weeks.

GC elevation in response to stress began immediately after animal immobilization and peaked approximately 30 min later. If immobilization was continued after the peak, the GC levels showed a gradual decrease over longer times [44,45]. Therefore, to use changes in GC levels as indicators of stress, the GC data were obtained 30 min after the beginning of the stress treatment.

All animal experiments were carried out in accordance with the Guiding Principles for the Care and Use of Research Animals of Obihiro University of Agriculture and Veterinary Medicine, Japan. The protocol was approved by the Committee on the Ethics of Animal Experiments of Obihiro University of Agriculture and Veterinary Medicine (Permit numbers: 25–155, 28–152, 29–164, 18–95, 19–162).

### 2.2. EF Exposure System

**Figure 1:**
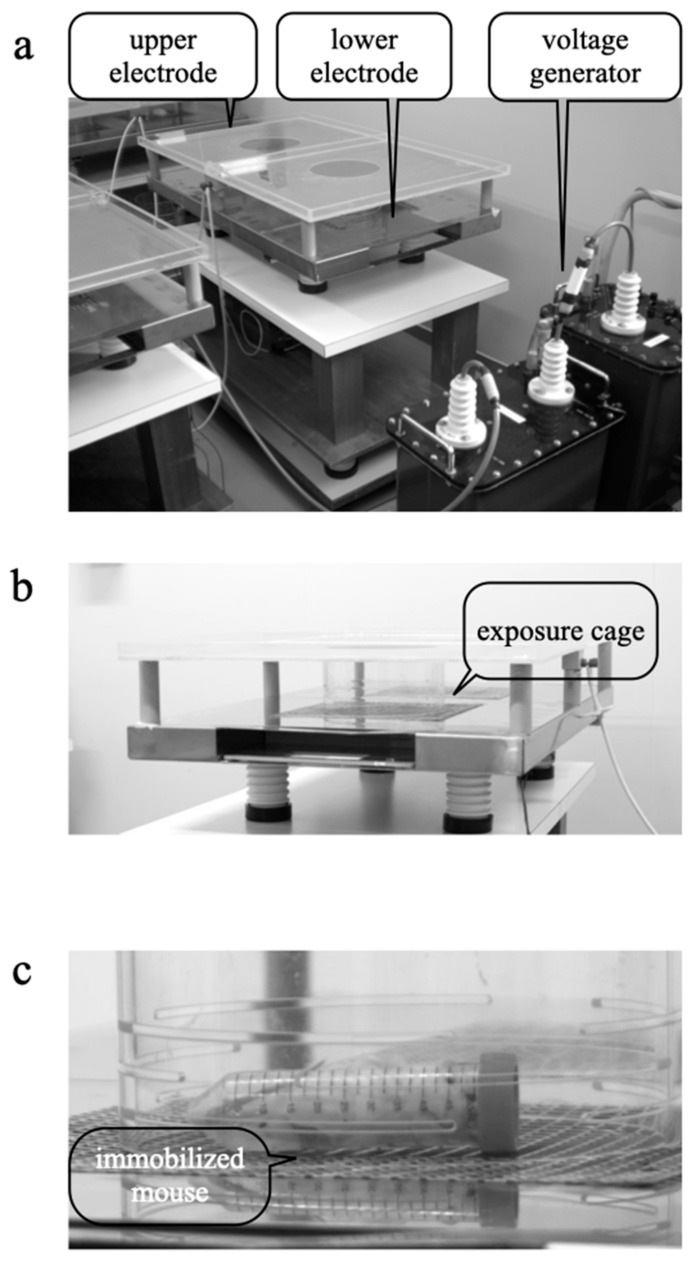
Electric field exposure system. (**a**) Voltage generator and parallel plate electrodes are shown. (**b**) Exposure cage had 100 mm × 5 mm slits spaced at 5 mm intervals. (**c**) A 50 mL centrifuge tube was used to immobilize the mice.

The EF exposure system consisted of three major parts: a high-voltage transformer unit (maximum output voltage: 30 kV; Hakuju, Tokyo, Japan), a constant-voltage unit (CVFT1-200H; Tokyo Seiden, Tokyo, Japan) to avoid unexpected interference from electrical noise originating from the commercial power supply, and an EF exposure cage [22,33,35]. The cylindrical plastic cage (diameter, 200 mm; height, 100 mm) had two stainless steel electrodes (1000 × 600 mm) placed above and below it (Figure 1a,b). The cylindrical cage had slits (length, 100 mm; width, 5 mm) spaced at 5 mm intervals (Figure 1b) to prevent the effects of smudges (from feces or saliva) on the formation of a stable EF. A separate cage and tube were used for each animal and were reused after being washed with a neutral detergent and completely dried. A digital thermometer placed on the lower electrode measured the temperature before EF exposure and 10, 20, 30, 40, 50, and 60 min after EF exposure. The temperature inside the cage was 25 ± 3 °C during the experimental period and did not change. The humidity was maintained between 45% and 55%.

Mice in the EF-treated groups were exposed to an EF of 10 kV/m for 60 min generated by applying 50 Hz, 1 kV to the upper electrode, and grounding the lower electrode. An optical fiber voltmeter, which measures EF intensity by the Pockels effect, an electro-optic voltage sensor attached with a two-cored Bi_12_SiO_20_ fiber (FOVM 03; Sumitomo Electric, Osaka, Japan), and a digital multimeter (Fluke 87; Fluke, Everett, WA, USA) were used to measure the field intensity and verify the system’s operation. The EF intensity was measured at 273 arbitrary points (21 × 13) on each cage floor. The 10 kV/m EF intensity applied to the cage had a margin of error of ±4% (outside the exposure cage) and ±0.1% (inside). In addition, EF distribution around the mouse was estimated by simulation software OpenSTF Version 1.6.0 (EEM, Saitama, Japan). A homogeneous ellipsoid or sphere model was used in our simulation. The permittivity was regarded as infinity because the model was treated as a homogeneous ideal conductor. The finite difference method (FDM) was used in the software. FDM is one of the numerical calculation methods for solving differential equations, where finite difference approximations of derivatives are made.

To measure the MF intensity at the area in which each mouse was exposed to the EF, a portable alternating current magnetic field meter (TMM-1; Electric Power Engineering Systems, Kanagawa, Japan) was used. The magnetic field intensity was approximately 0.012 ± 0.004 μT when a 50 Hz and 10-kV/m EF was generated in the space.

### 2.3. Immobilization Stress

Mice in the control group were housed in the EF cage for 60 min without exposure to an EF (i.e., 0 V/m). Stress was induced by immobilization of each mouse separately inside a 50 mL centrifuge tube (Nippon Genetics, Tokyo, Japan), which was placed on the lower electrode (Figure 1c) [22,33,35]. The centrifuge tube for restraint has a slit in it to enable the mouse from breathing, and the lid has a hole that is about 3 mm in diameter.

### 2.4. Experiment 1: Effect of EF on Plasma Glucocorticoid Levels in Repeatedly Immobilized Mice

**Figure 2:**
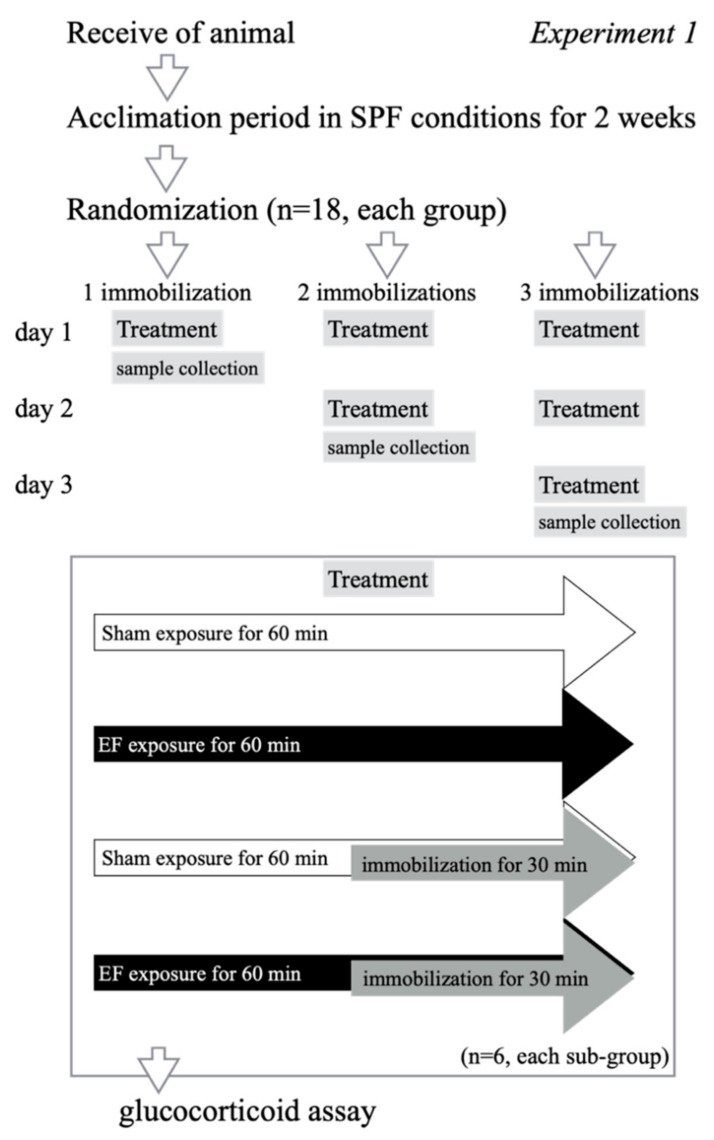
Experimental design to assess the effect of EF on plasma glucocorticoid levels in repeatedly immobilized mice, Experiment 1. To examine the effect of an EF on plasma glucocorticoids in repeatedly immobilized mice, 54 mice were divided into three groups according to the number of stress episodes (one, two, or three immobilizations; *n* = 18 mice each). Each group was further divided into three subgroups (*n* = 6 per subgroup): a control (stress (−)/EF (−)), an immobilization-alone (stress (+)/EF (−)), and a co-treatment group (stress (+)/EF (+)). Mice in the co-treatment group were exposed to the EF for 60 min and were immobilized during the second half (30 min) of the EF exposure period. In the control group, mice were handled in an identical manner, except that the EF condition was 0 V/m, and they were not immobilized.

Seventy-two mice were divided into three groups based on the number of stress episodes: one, two, or three immobilizations. Each group of 24 mice was divided into the following four subgroups (*n* = 6 per subgroup): the sham exposure, no immobilization (stress (−)/EF (−)), EF-alone (stress (−)/EF (+)), immobilization-alone (stress (+)/EF (−)), and co-treatment (stress (+)/EF (+)) (Figure 2). Mice in the co-treatment groups were exposed to 50 Hz and 10 kV/m for 60 min and were immobilized during the second half (30 min) of the EF exposure period. The rationale for the 10 kV/m setting is based on the results of previous experiments comparing the stress-reducing effects of different field strengths [33]. Mice in the sham exposure, no immobilization group (stress (−)/EF (−)) were housed in the EF cage for 60 min without exposure to an EF (i.e., 0 V/m). Plasma GC levels were measured at 60 min after the initiation of the EF. For example, in the group with three repeated immobilizations, mice were immobilized once a day for 3 consecutive days, and blood was collected after the third day of immobilization.

### 2.5. Experiment 2: Effect of the Polarization of the Electrode on the Suppressive Effect of EF

**Figure 3:**
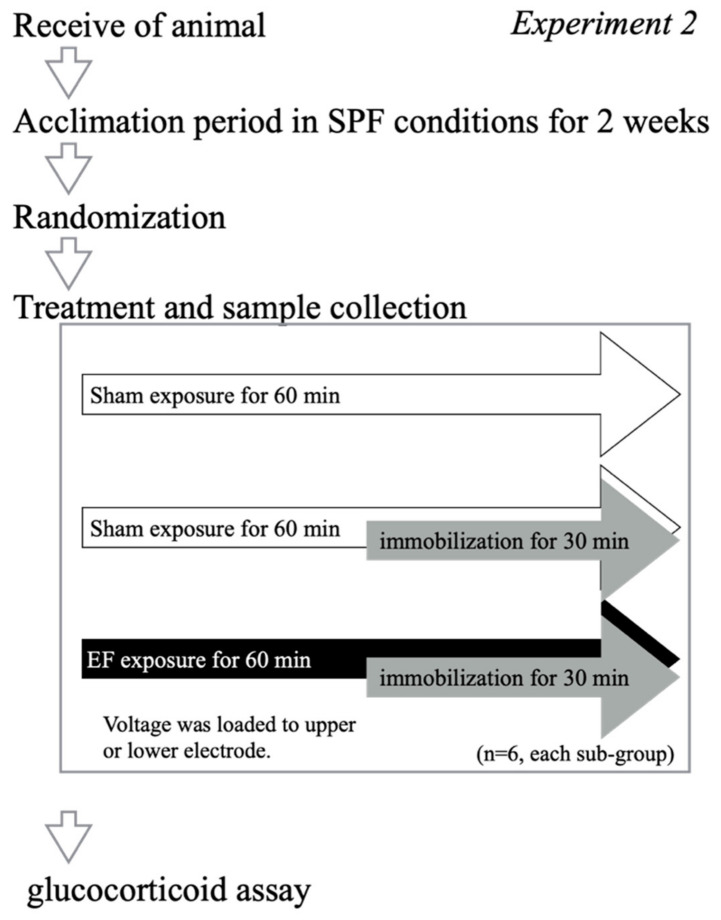
Experimental design to assess the effect of the polarization of the electrode on the suppressive effect of EF, Experiment 2. To examine the effect of the polarization of the electrode that the high voltage signal was loaded into on the suppressive effect of an EF on plasma GC in immobilized mice, 24 mice were divided into four groups (*n* = 6, each group): control (stress (−)/EF (−)), immobilization-alone (stress (+)/EF (−)), and two co-treatment groups (stress (+)/EF (+)), in which a voltage of 1 kV was applied to either the upper or lower electrode at 10 kV/m.

Twenty-four mice were divided into four groups (*n* = 6, each group): sham exposure, no immobilization (stress (−)/EF (−)), immobilization-alone (stress (+)/EF (−)), and two co-treatment groups (stress (+)/EF (+)) in which a voltage of 1 kV was applied to either the upper or lower electrode at 10 kV/m (Figure 3). Plasma GC levels were measured at 60 min after the initiation of the EF.

### 2.6. Experiment 3: Effect of EF Shield on the Suppressive Effect of EF

**Figure 4:**
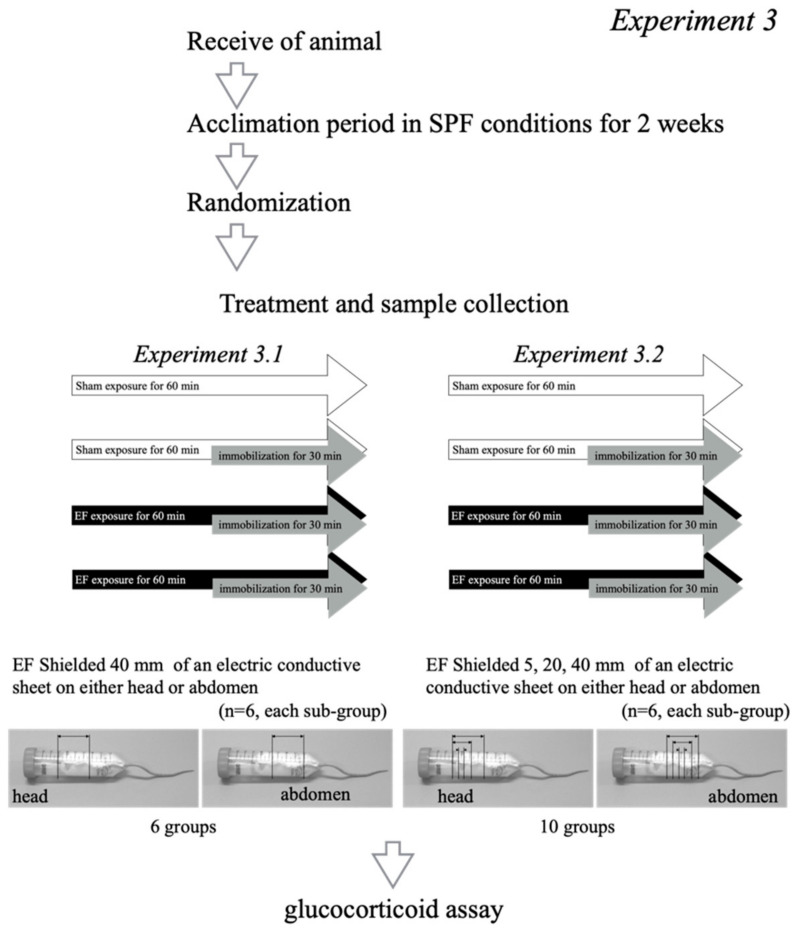
Experimental design to assess the effect of EF shield on the suppressive effect of EF, Experiment 3. To examine the effect of an EF shield on the suppressive effect of an EF on plasma GC, 96 mice were divided into 36 mice (6 groups) and 60 mice (10 groups). Thirty-six mice were divided into 6 groups (*n* = 6 each group): control (stress (−)/EF (−)), EF-alone (stress (−)/EF (+)), immobilization-alone (stress (+)/EF (−)), co-treatment (stress (+)/EF (+)), and co-treatment with shielding at the head, or at the abdomen, using a 40 mm–long shield sheet (stress (+)/EF (+)/shielded) (Figure 4). Subsequently, 60 mice were divided into 10 groups (*n* = 6 each group): control (stress (−)/EF (−)), EF-alone (stress (−)/EF (+)), immobilization-alone (stress (+)/EF (−)), co-treatment (stress (+)/EF (+)), and co-treatment with shielding at the head, or at the abdomen, using different length shield sheets (stress (+)/EF (+)/shielded):control (stress (−)/EF (−)), EF-alone (stress (−)/EF (+)), immobilization-alone (stress (+)/EF (−)), co-treatment (stress (+)/EF (+)), and co-treatment with shielding to the head or the abdomen (stress (+)/EF (+)/shielded (sheet width: 5, 20, or 40 mm on either head or abdomen)).

Experiment 3 consisted of two experiments. To test whether the shielding of EF cancels the effect of EF on the GC response, 36 mice were divided into 6 groups (*n* = 6 each group): sham exposure, no immobilization (stress (−)/EF (−)), EF-alone (stress (−)/EF (+)), immobilization-alone (stress (+)/EF (−)), co-treatment (stress (+)/EF (+)), and co-treatment with shielding at the head or the abdomen, using a 40 mm–long shield sheet (stress (+)/EF (+)/shielded) (Experiment 3.1, Figure 4). Subsequently, to evaluate the effect of the EF on the GC response when shielding length was changed, 60 mice were divided into 10 groups (*n* = 6 each group): control (stress (−)/EF (−)), EF-alone (stress (−)/EF (+)), immobilization-alone (stress (+)/EF (−)), co-treatment (stress (+)/EF (+)), and co-treatment with shielding at the head, or at the abdomen, using different length shield sheets, 5–40 mm, (stress (+)/EF (+)/shielded) (Figure 4). The mice in the co-shielded groups were wrapped with a polypropylene sheet EF shield (0.1 mm thick and 5–40 mm width) (TW-CLF-CL; Tanimura, Nara, Japan) at either the head or the abdomen (Experiment 3.2, Figure 4). Various widths of EF shields allowed for the evaluation of the effect of shields at different areas of the mice. Plasma GC levels were measured 60 min after the initiation of the EF.

### 2.7. Plasma Glucocorticoid Level

All EF treatments and blood-collection procedures were completed between 10:00 and 12:30 to decrease the risk of interrupting the circadian rhythms of the mice. The experimental day and time slot of the day were randomized within each group. Mice were subjected to a test in one day, and the mice were divided into 3 or 4 pairs for treatments. Within 8 min after EF treatment, 800 µL of blood was collected through the cardiac puncture into a heparinized tube from each mouse, using 3% isoflurane anesthesia (Mylan, Tokyo, Japan). Ten microliters of the sample was diluted in a sheath fluid (MEK-640, Nihon Kohden, Tokyo, Japan) for the analysis of the white blood cell count, red blood cell count, hemoglobin, hematocrit, mean cell volume, mean cell hemoglobin, and platelet count, using a Celltak system (Nihon Kohden). The remainder of the sample was centrifuged at 1500× *g* for 10 min at 4 °C, and the plasma was collected and stored at −80 °C until use.

To measure the plasma GC level, 200 μL of plasma was mixed with 900 µL of isooctane (2,2,4-trimethylpentane; Wako, Osaka, Japan), vortexed, and centrifuged at 380× *g* for 5 min at 24 °C. The upper layer was discarded, and 900 µL of chloroform (Wako) was added to the lower layer. The sample was vortexed and centrifuged at 380× *g* for 5 min at 24 °C. The upper and white membranous layers were removed, and 800 µL of the lower layer was transferred to a new tube and mixed with 320 µL of a 65% concentrated sulfuric acid:35% ethanol solution (Wako) to measure total GC by using sulfuric acid–induced fluorescence of GC [46]. The solution was vortexed and then incubated in the dark for 3.5 h; the fluorescence intensity of the sample was measured at 519 nm, with excitation at 475 nm, using a spectrofluorophotometer (RF-5300PC; Shimadzu, Kyoto, Japan).

### 2.8. Experiment 4: Change in Bodyweight

Twenty-eight mice were divided into four groups (*n* = 7 per group): control group (stress (−)/EF (−)), EF-alone (stress (−)/EF (+)), immobilization-alone group (stress (+)/EF (−)), and co-treatment group (stress (+)/EF (+)), in which a voltage of 1 kV was applied to the upper electrode. The body weight of each mouse was measured on days 1, 4, and 7–19, and the mice underwent immobilization or immobilization with EF on day 12.

### 2.9. Statistical Analysis

Differences between groups were evaluated by using a one-way, two-way, or three-way analysis of variance (ANOVA), and differences between two groups were evaluated by using post hoc analyses for Experiments 1, 2, and 3. For Experiment 3, the correlation and regression were assessed by using slope regression analysis, respectively. For Experiment 4, repeated-measures ANOVA was used for analysis. Statistical significance was defined as *p* < 0.05. Furthermore, all statistical analyses were conducted by using Prism Version 8 (GraphPad Software, La Jolla, CA, USA).

## 3. Results

### 3.1. Experiment 1: Effect of EF on Plasma Glucocorticoid Levels in Repeatedly Immobilized Mice

**Figure 5:**
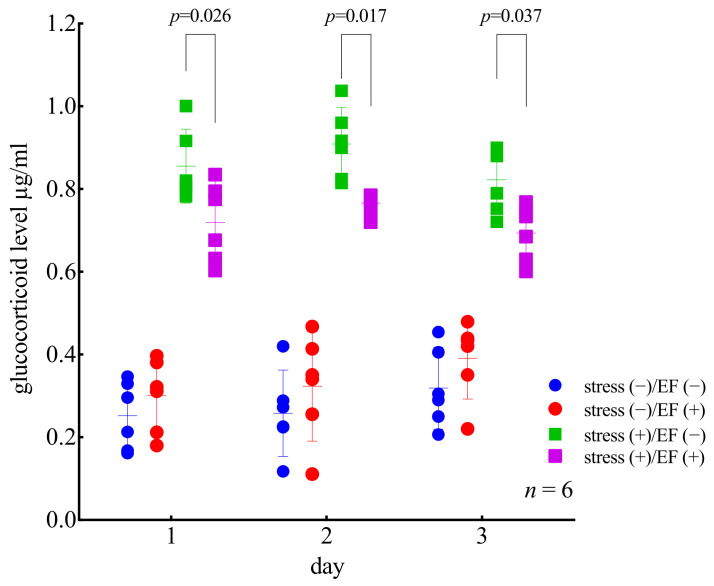
Effect of repeated immobilization on plasma GC level and effect of EF on the immobilization-induced increase in plasma GC level, Experiment 1. As determined using a three-way ANOVA, for the main effect, for “day”, F (DFn, DFd) = F (2, 60) = 0.8763, *p* = 0.4216. The effect is therefore considered not significant. For “stress”, F (DFn, DFd) = F (1, 60) = 554.2, *p* < 0.0001. For “sham/EF”, F (DFn, DFd) = F (1, 60) = 3.193, *p* = 0.079. For the “day × stress” interaction, F (DFn, DFd) = F (2, 60) = 4.336, *p* = 0.0174. The effect is therefore considered significant. For “day × sham/EF”, F (2, 60) = 0.05075, *p* = 0.9506; for “stress × Sham/EF”, F (1, 60) = 22.80, *p* < 0.0001; and for “day × stress × Sham/EF”, F (2, 60) = 0.03141, *p* = 0.9691. Two-way ANOVA (“day” × “group”), for the main effect, “treatment”, F (DFn, DFd) = F (3, 60) = 193, the *p*-value is < 0.0001, the effect is considered significant and “day”, F (DFn, DFd) = F (2, 60) = 0.876, the *p*-value = 0.4215. For the interaction, F (DFn, DFd) = F (6, 60) = 1.47, the *p*-value = 0.2029. Subsequently, Dunnett’s multiple comparisons test indicated that the *p*-value between stress (+)/EF (−) and stress (+)/EF (+) is 0.0259 on day 1, 0.017 on day 2, and 0.0371 on day 3. The plasma GC levels were significantly lower in mice who were immobilized and exposed to the EF than in mice who were immobilized but not exposed to the EF, regardless of the number of immobilizations. Mice who were not immobilized had significantly lower levels of plasma GC than those of the other groups.

In order to analyze the main effects and their interactions, ANOVA was conducted for “stress”, “sham/EF”, and “days”. The effect of “stress” was significant, but no significant differences were detected for the other two factors (*p* < 0.0001; three-way ANOVA, Figure 5). The interaction was significant for “stress and days” (*p* = 0.0174) and “stress and sham/EF” (*p* < 0.0001). The plasma GC levels were significantly different between mice who underwent different treatments (*p* < 0.0001; two-way ANOVA and Dunnett’s multiple comparisons test); however, they were not significantly different between mice treated on different days (Figure 5). The GC levels in the stress (+)/EF (−) group were significantly higher than those in the stress (−)/EF (−) group for mice who underwent one, two, and three days of immobilization (*p* < 0.0001; Dunnett’s multiple comparisons test; Figure 5). The GC levels in the stress (+)/EF (+) group were significantly lower than those in the stress (+)/EF (−) group (*p* = 0.02591 for one immobilization; *p* = 0.017 for two immobilizations; *p* = 0.0371 for three immobilizations; Figure 5). The behavior of mice during restraint, motionlessness, and gnawing, and turning their bodies at the slit part of the restraint device were observed, but no noticeable changes were found during repeated restraint and EF treatment.

### 3.2. Experiment 2: Effect of the Polarization of the Electrode on the Suppressive Effect of EF

**Figure 6:**
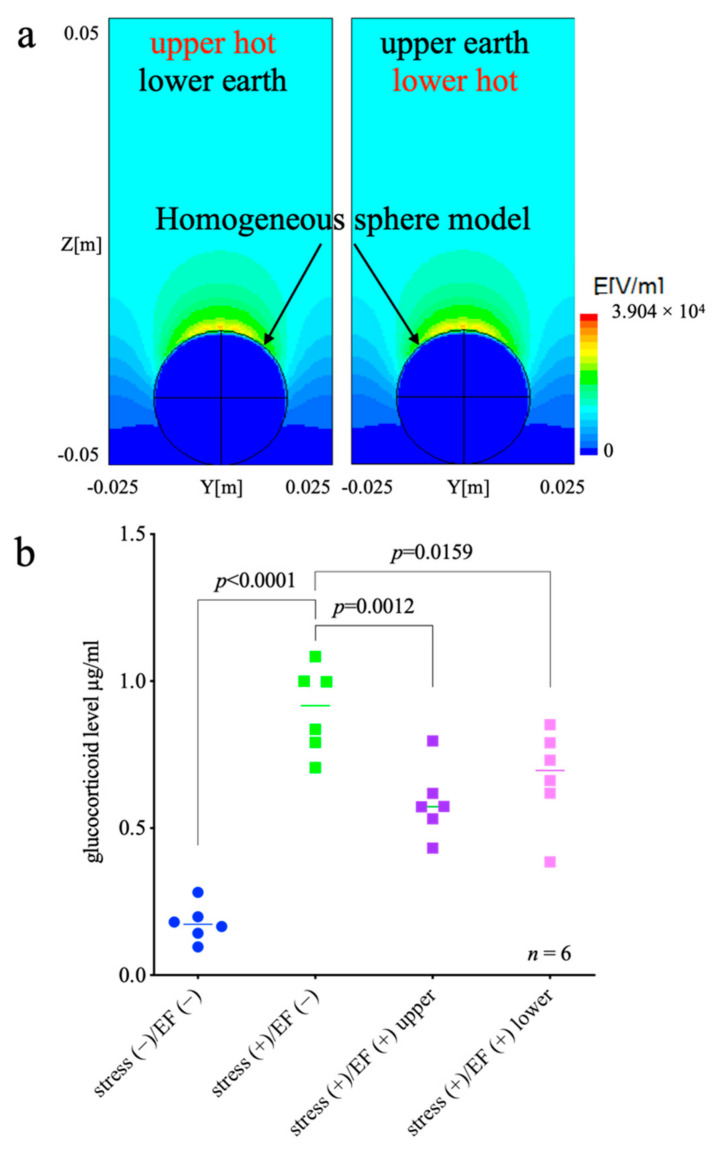
Effect of the polarization of the electrode on the suppressive effect of EF, Experiment 2. (**a**) The distribution of the electric field (EF) on the mouse’s body. (**b**) One-way ANOVA, F (DFn, DFd) = F (3, 20) = 32.97, the *p*-value < 0.0001. Subsequently, Dunnett’s multiple comparisons test indicated that the *p*-value between stress (+)/EF (−) vs. stress (+)/EF (+) upper is 0.0012, and the *p*-value between stress (+)/EF (−) vs. stress (+)/EF (+) lower is 0.0159. The EF was found to reduce the immobilization-induced increase in GC levels in immobilized mice, regardless of which electrode EF was loaded with.

The EF distribution surrounding a mouse exposed to EF is shown in Figure 6a. In the upper half of the region, the EF is relatively homogeneous; the EF at 50 mm in height in the centerline is 13.4 kV/m and 6% stronger than that at 100 mm height. In contrast, EF is concentrated in the lower half of the region around the mouse model. The EF at the top of the mouse model is 39 kV/m and 3.1 times higher than that at 100 mm height. Those EF distributions are almost identical regardless of the electrode polarization, i.e., the difference is 10^−6^. In addition, the GC levels in the stress (+)/EF (−) group were significantly higher than those in the stress (−)/EF (−) group (*p* < 0.0001; one-way ANOVA and Dunnett’s multiple comparisons test; Figure 6b). The GC levels in both stress (+)/EF (+) groups were lower than those in the stress (+)/EF (−) group, regardless of whether the signal was loaded into the upper or lower electrode (*p* = 0.0012 and 0.0159, respectively, Figure 6b).

### 3.3. Experiment 3: Effect of EF Shield on the Suppressive Effect of EF

**Figure 7:**
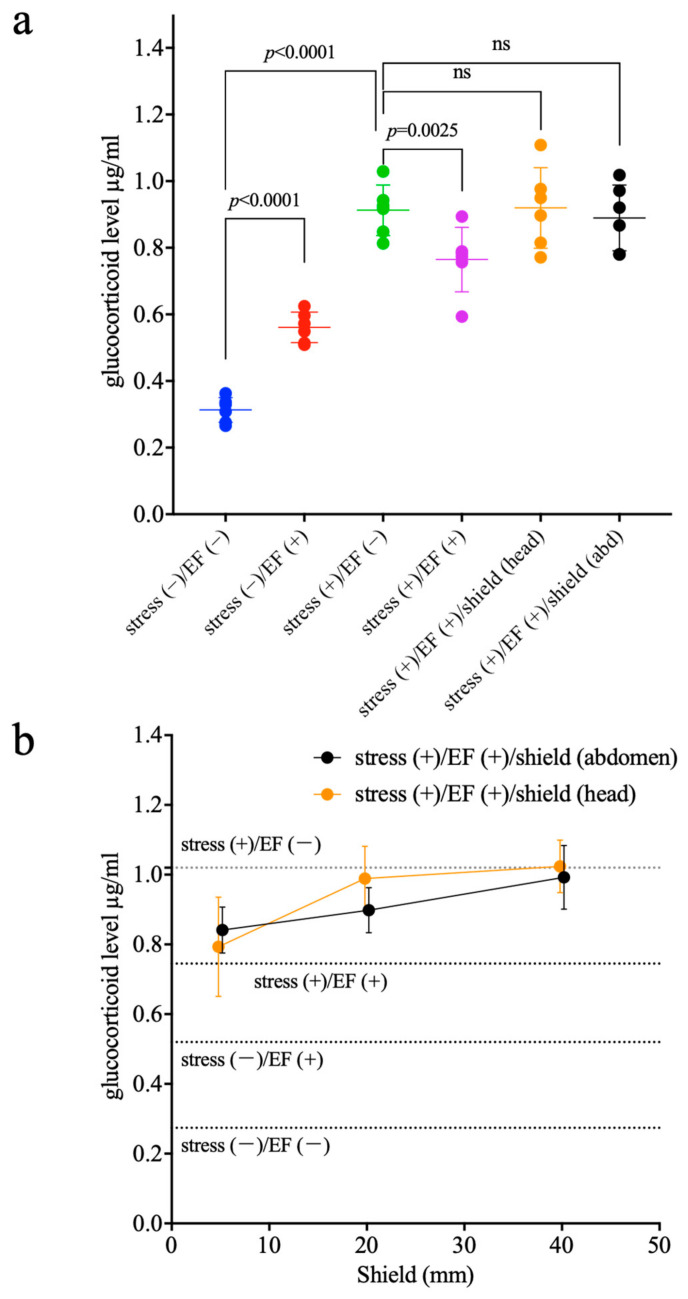
Effect of immobilization on plasma GC level and effect of EF on the immobilization-induced increase in plasma GC level when the mouse was shielded from EF at the head or abdomen, Experiment 3. (**a**) Two-way ANOVA (stress (−)/EF (−), stress (−)/EF (+), stress (+)/EF (−) and stress (+)/EF (+)): for the main effect, “EF or sham”, F (DFn, DFd) = F (1, 20) = 3.216, the *p*-value = 0.0881; and “stress”, F (DFn, DFd) = F (1, 20) = 207.7, the *p*-value < 0.0001. For the interaction, F (DFn, DFd) = F (1, 20) = 50.39, the *p*-value < 0.0001. Subsequently, Bonferroni’s multiple comparisons test indicated the *p*-value between EF (−) vs. EF (+) is 0.0025 in “stress (+)” and is < 0.0001 in “stress (−)”. One-way ANOVA (stress (−)/EF (−), stress (−)/EF (+), stress (+)/EF (−), stress (+)/EF (+), stress (+)/EF (+)/shield (head) and stress (+)/EF (+)/shield (abdomen)), F (DFn, DFd) = F (4, 25) = 48.59, the *p*-value < 0.0001. Subsequently, Dunnett’s multiple comparisons test indicated that the *p*-value between stress (+)/EF (−) vs. stress (+)/EF (+)/shield (head) and stress (+)/EF (+)/shield (abdomen) is not significant (ns). Reduction of immobilization-induced increases in GC was not found in mice with EF shields. (**b**) Two-way ANOVA (stress (−)/EF (−), stress (−)/EF (+), stress (+)/EF (−) and stress (+)/EF (+)): for the main effect, “EF or sham”, F (DFn, DFd) = F (1, 20) = 1.696, the *p*-value = 0.2079; and “stress”, F (DFn, DFd) = F (1, 20) = 150.8, the *p*-value < 0.0001. For the interaction, F (DFn, DFd) = F (1, 20) = 35.32, the *p*-value < 0.0001. Subsequently, Bonferroni’s multiple comparisons test indicated that the *p*-value between EF (−) vs. EF (+) is 0.0005 in “stress (+)” and is < 0.0001 in “stress (−)”. Two-way ANOVA, ((stress (+)/EF (+)/shield (head)) or (stress (+)/EF (+)/shield (abdomen))) vs. (shield length (5 mm, 20 mm, 40 mm)): for the main effect, “shield length”, F (DFn, DFd) = F (2, 30) = 13.22, the *p*-value is < 0.0001; and “shielded portion”, F (DFn, DFd) = F (1, 30) = 0.6422, the *p*-value = 0.4292; for the interaction, F (DFn, DFd) = F (2, 30) = 1.690, the *p*-value = 0.2017. Simple regression analysis, F (DFn, DFd) = F (1, 32) = 0.779, the *p*-value = 0.384.

Two-way ANOVA results showed that the factor of immobilization significantly affected the change in GC levels (*p* < 0.0001; two-way ANOVA; Figure 7a), whereas the factor of EF treatment was not found to have any effect (*p* = 0.0881). On the other hand, an interaction was observed (*p* < 0.0001). Bonferroni’s multiple comparison test showed that the GC level in the stress (−)/EF (+) group was higher than those in the stress (−)/EF (−) group (*p* < 0.0001), and the GC level of the stress (+)/EF (+) group was lower than that of the stress (+)/EF (−) group (*p* = 0.0025, Figure 7a). One-way ANOVA showed that the GC levels of the stress (+)/EF (+) group shielded from EF by a 40 mm–wide insulator were not significantly different from those of the stress (+)/EF (−) group (Figure 7a).

For Experiment 3.2 (Figure 7b), the results of two-way ANOVA showed that the factor of immobilization significantly affected the change in GC levels (*p* < 0.0001; two-way ANOVA; Figure 7b), whereas the factor of EF treatment did not appear to have a significant effect (*p* = 0.2079). On the other hand, an interaction was observed (*p* < 0.0001). Bonferroni’s multiple comparison test showed that the GC levels in the stress (−)/EF (+) group were higher than those in the stress (−)/EF (−) group (*p* < 0.0001), and the GC level of the stress (+)/EF (+) group was lower than that of the stress (+)/EF (−) group (*p* = 0.0005, Figure 7b).

The analysis of the effect of the insulator width factor by two-way ANOVA showed that the difference in insulator width significantly affected the change in GC levels (*p* < 0.0001); however, the factor when the insulator was shielded was not significant (*p* = 0.4292) and no interaction was found (*p* = 0.2017).

The regression analysis for the upper and lower body showed no difference in the slopes of the two (Y = 0.004355X + 0.8164 (head), Y = 0.006326X + 0.7984 (abdomen), *p* = 0.384, regression analysis). In the graph, there was a greater degree of inhibition of the effect of EF treatment on the GC response when the head was shielded; however, a two-way ANOVA and regression analysis showed no significant difference (20 mm, Figure 7b).

### 3.4. Experiment 4: Change in Bodyweight

**Figure 8:**
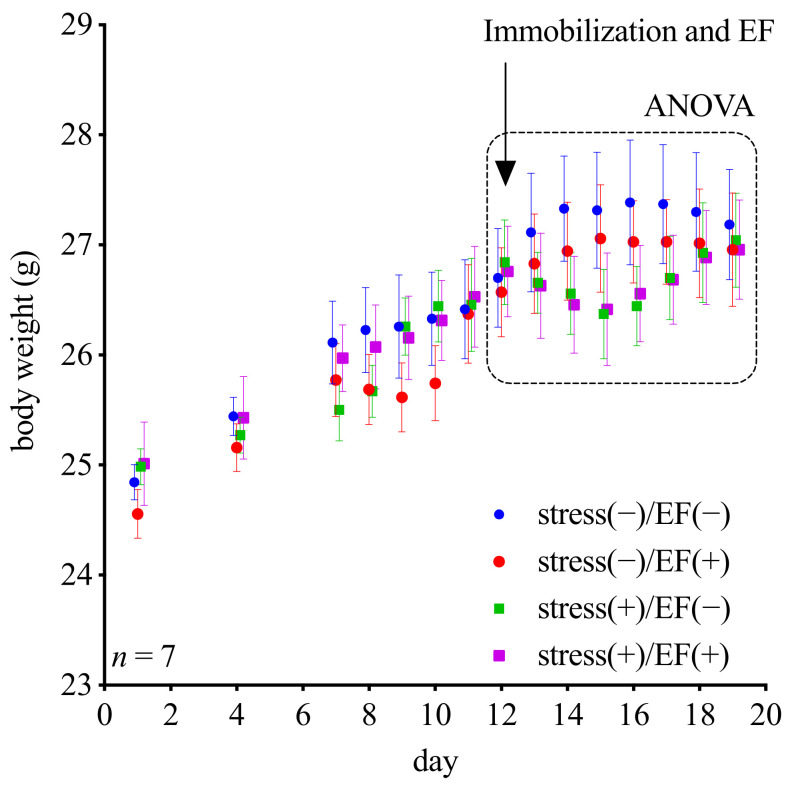
Changes in bodyweight, Experiment 4. The body weights of mice in the control group increased during the 19-day testing period. Those of mice in the immobilization-alone and co-treatment groups decreased for three days after immobilization occurred on the 12th day. For days 12 to 19 (see dashed box), two-way ANOVA (stress (−)/EF (−), stress (−)/EF (+), stress (+)/EF (−), and stress (+)/EF (+)) was performed. For the main effect, “treatment”, F (DFn, DFd) = F (3, 24) = 0.3280, the *p*-value is 0.8051; and “day”, F (DFn, DFd) = F (3.592, 86.20) = 5.567, the *p*-value = 0.0008, for the interaction between treatment and day, F (DFn, DFd) = F (21, 168) = 3.921, the *p*-value < 0.0001.

The body weights of mice in the stress (−)/EF (−) and the stress (−)/EF (+) groups increased during the testing period; however, those of mice in the stress (+)/EF (−) and stress (+)/EF (+) groups decreased for three days after immobilization occurred on the 12th day. However, according to the ANOVA results, there was no statistical difference among all the groups (Figure 8).

## 4. Discussion

In the present study, we determined the biological effects of ELF-EF in immobilized mice. We measured the increase in plasma GC levels as an index of stress. We studied the relationship of the stress-reducing effect of ELF-EF with repeated immobilization, the polarization of the electrode, and the use of an EF shield. We also examined the effects of ELF-EF on the growth of mice with or without immobilization.

We found that the plasma GC levels in immobilized mice were approximately 3.5-fold higher than those in the mice that were not subjected to stress, suggesting that the immobilization procedure activated the endocrine system of the pituitary–adrenocortical axis and/or the sympathetic-adrenomedullary system [31,46,47]. In mice that underwent immobilization stress and were also exposed to an EF of 50 Hz, the increase in the plasma GC levels was lower than that in mice that were immobilized but not exposed to an EF (Figure 5). The current study revealed that EF exposure has an anti-stress-like effect for single stress and repeated stresses, using the stress-reduction model. Tachyphylaxis of the effect of the EF was not observed among immobilizations repeated three times (Figure 5). Regarding the inhibitory effect of ELF-EF on the GC response, no study has been conducted to confirm the conditions that cause tachyphylaxis. Moreover, there has been no study in which animals were exposed to ELF-EF for more than 1 h per day while using the experimental system. In the current study, EF treatment for 1 h per day was repeated for 3 days, which is not chronic exposure. Therefore, it is necessary to investigate the effects of uninterrupted prolonged exposure for several hours to several days in future studies. In contrast, with prolonged exposure, there is a problem that the level of EF to which the living body is exposed decays over time due to contamination by urine and feces of the test animals; therefore, it is necessary to find a way to resolve the issue. However, the experimental system in this study can capture the EF effects because the duration from the field treatment or restriction until sampling was relatively short, 30–60 min, which is a trade-off when conducting experiments with long-term exposure of field.

Additionally, in our previous study, when C57BL/6J male mice were exposed to a 50-Hz EF for 30 min per day for 11 days and placed in a cage with a superovulated female of the same strain, the successful copulation rate of males was significantly improved compared with that of males that were not exposed to an EF [29]. The effect reduced as the period of EF treatment decreased. A stress-induced increase in adrenal GC levels causes an increase in orthologous neuropeptides known as RFamide-related peptides that contribute to the hypothalamic suppression of reproductive functions [37]. Thus, the improvement of the copulation rate in C57BL/6J mice [29] may be related to the EF-induced modulation of the GC level. The bone density of human males exposed to EF therapy is reportedly higher than that of age-matched males, and the bone density increased as the duration of EF treatment increased [21]. Glucocorticoid-induced osteoporosis [43] may be suppressed by the EF-induced modulation of GC levels [21,29].

In addition, ELF-EF has been found to be effective for alleviating the pain of musculoskeletal or for the undefined origin and insomnia [8,21]. It is well-known that GC modifies inflammation [38,39,40,41,42], and the effect of ELF-EF on stress and tissue damage involving inflammatory events has been suggested. As the relationship between plasma GC levels and ELF-EF has been elucidated, more medical applications of ELF-EF may be discovered.

There have been reports of a correlation between EF exposure and biological response or its change, which was considered to be a biological effect of EF exposure, but it was later discovered to be caused by other factors. It has been reported that electric shock from a short circuit between a charged mouse and the exposure system could provoke a stress response in the animal [48]. In this study, we observed that the polarization of the electrode does not affect the suppression of stress-related responses in immobilized mice (Figure 6b). The finding is consistent with the simulation that EF distribution around the ellipsoid model is not influenced by electrode polarity (Figure 6a). Theoretically, EF formed between the electrodes in a parallel plate electrode system should be the same irrespective of the electrodes from which the voltage is applied. However, as mentioned above, unexpected factors may affect the results of the experiment; thus, this study was planned. Moreover, the position of electrodes would be one of the important specifications in considering the medical applications of EF, and the results of this study provide basic information for controlling or assessing the biological effects of EF by electrode placement in the future. In Experiment 2, one group (stress (−)/EF (+)) is not shown. Because of the deficiencies in this group, we could not study whether EF exposure had an effect on glucocorticoid levels.

The suppressive effect of EF to GC response was reduced by an EF shield (Figure 7a), indicating that the suppressive effect of EF is dependent on the intensity distribution of the EF over the exposed regions of the body, even when the EF strength remained unchanged, as is consistent with the findings of a previous study [35]. The result that shielding the head from EF might have a more attenuated stress-inhibitory effect than that of shielding the abdomen suggests that the biological effect of EF on the head is greater than that on the other portions (Figure 7b). If this speculation was correct, any structure, including the sensory organs (eyes, nose, and ears) in the head, both inside and outside the skull, could be considered as a possible target. It has been reported that some cell receptors exhibit sensitivity to EMF [49]. We observed the effects of ELF-EF on [Ca^2+^]_i_ of a protein that senses extracellular Ca^2+^ concentrations in calcium-sensing receptors [50]. Differences in the expression of these receptors in the head and abdomen can also contribute to this result. However, it has been reported that the penetration of the ELF-EF into the living body is extremely low [2,3], and data regarding the effect of ELF-EF at a cellular level are limited. On the other hand, assuming that the effect of EF-distribution on the body surface acts more strongly as a mechanism than the current induced in the body in power frequency ELF-EF [20], the present observations may be said to be consistent.

We determined the changes in the bodyweight of mice that were immobilized, as well as mice that were both immobilized and exposed to EF. Mice that were immobilized lost approximately 5% of their bodyweight within 3 days after the treatment (Figure 8), regardless of the presence or absence of EF. The loss of bodyweight occurred despite the fact that we also observed a suppressive effect of the increase in GC in mice exposed to EF. After 7 days, all mice recovered their lost bodyweight in the graph, suggesting that the ELF-EF did not adversely affect the growth of mice. These results indicate that the recovery of growth was not affected as a result of ELF-EF lowering the threshold of GC required to trigger the GC response. The high intensity of stress (immobilization) may account for the discrepancy of the effect of EF in GC elevation suppression and changes in bodyweight. According to the ANOVA results, there was no statistical difference among all the groups. Therefore, additional studies on this aspect, especially with an increased sample size, would be necessary.

The results of our study are consistent with those of other studies, indicating the robustness of this animal model to assess the efficacy of biological mechanisms induced by EF exposure (Figure 5, Figure 6b, and Figure 7). However, our experimental model is not without limitations. In three experiments, an EF intensity of 10 kV/m was selected based on previous studies that assessed EF intensities ranging from 2.5 to 200 kV/m, where 10 kV/m showed the highest inhibitory effect of EF on the GC response [33]. An additional reason for selecting this EF condition was that EF at an intensity greater than 50 kV/m generated vibration and/or noise [33]. The effects of vibrations and/or noise were difficult to distinguish from those of the EF and could not be excluded as a possible artifact at higher intensities. The possibility of artifacts needs to be addressed and resolved so that higher EF intensities can be further investigated. This finding is consistent with the effect of ELF-EF. However, further research is required to determine the effect of ELF-EF in human studies. Furthermore, in this study, total glucocorticoids were evaluated, but not their fractions. This is one of the limitations of this study and previous studies [22,26,27,28,29,30,31,32,33]. In our previous study, we mentioned a similar pattern for corticosterone and deoxycorticosterone, i.e., higher levels in the restraint group compared with the unrestrained group, and slightly lower levels in the combined treatment group [22]. It should be considered that the above viewpoint is a future challenge in understanding the mechanism of biological effects of ELF-EF. Meanwhile, the evaluation system using total glucocorticoids as an index seems to be fairly robust in understanding the relationship between the ways ELF-EF are treated, including electrode placement to control the distribution of ELF-EF on the body surface, and their biological effects.

The balance between the health risks and medical applications of ELF-EF remains controversial. Both ICNIRP and WHO have reported that the health risks are limited, although further research is required. However, biological changes due to ELF-EF cannot be ignored, no matter how small they appear to be. Although we focused on the effect of EF on the elicited GC response in mice, it is also necessary to carefully examine whether the EF itself plays a role as a stressor.

## 5. Conclusions

The reproducibility of the effect of ELF-EF on plasma GC level by the system was confirmed in this study. The stress-reducing effect was observed not only after a single stress event but also after multiple stress events. The inhibitory effect of EF on the GC response will not rapidly disappear with repeated application. As the effect was not affected by the polarity of the electrodes, the possibility of EF being responsible for the observed stress-reducing effect was enhanced. It was also observed that the effect can be suppressed by shielding the EF, and the result indicates that EF treatment of the head may be more effective than that of the abdomen. There was no effect of ELF-EF on stress-induced weight loss and recovery observed. EF exposure under the condition of inhibitory effect on stress hormones does not have any negative effect on the stress-induced weight change of the organism. The evaluation system used in this study can qualitatively and quantitatively elucidate the exact biological effects of ELF-EF. The present study improves the understanding of the biological effect and medical applications of ELF-EF.

## Data Availability

We declare that all data supporting the findings of this study are available within the article.
